# Radiolysis Studies of Oxidation and Nitration of Tyrosine and Some Other Biological Targets by Peroxynitrite-Derived Radicals

**DOI:** 10.3390/ijms23031797

**Published:** 2022-02-04

**Authors:** Lisa K. Folkes, Silvina Bartesaghi, Madia Trujillo, Peter Wardman, Rafael Radi

**Affiliations:** 1 Formerly of the Gray Cancer Institute and MRC Oxford Institute for Radiation Oncology, University of Oxford, Oxford OX3 7DQ, UK; folkeslk@gmail.com (L.K.F.); peterwardman@btinternet.com (P.W.); 2 Departamento de Bioquímica, Facultad de Medicina, Universidad de la República, Montevideo 11800, Uruguay; sbartesa@fmed.edu.uy (S.B.); madiat@fmed.edu.uy (M.T.); 3 Centro de Investigaciones Biomédicas (CEINBIO), Universidad de la República, Montevideo 11800, Uruguay

**Keywords:** radiolysis, hydroxyl radicals, carbonate radicals, nitrogen dioxide radicals, tyrosine, dityrosine, nitric oxide, nitrotyrosine, lipoic acid, hydrogen sulfide, DMPO, desferrioxiamine

## Abstract

The widespread interest in free radicals in biology extends far beyond the effects of ionizing radiation, with recent attention largely focusing on reactions of free radicals derived from peroxynitrite (i.e., hydroxyl, nitrogen dioxide, and carbonate radicals). These radicals can easily be generated individually by reactions of radiolytically-produced radicals in aqueous solutions and their reactions can be monitored either in real time or by analysis of products. This review first describes the general principles of selective radical generation by radiolysis, the yields of individual species, the advantages and limitations of either pulsed or continuous radiolysis, and the quantitation of oxidizing power of radicals by electrode potentials. Some key reactions of peroxynitrite-derived radicals with potential biological targets are then discussed, including the characterization of reactions of tyrosine with a model alkoxyl radical, reactions of tyrosyl radicals with nitric oxide, and routes to nitrotyrosine formation. This is followed by a brief outline of studies involving the reactions of peroxynitrite-derived radicals with lipoic acid/dihydrolipoic acid, hydrogen sulphide, and the metal chelator desferrioxamine. For biological diagnostic probes such as ‘spin traps’ to be used with confidence, their reactivities with radical species have to be characterized, and the application of radiolysis methods in this context is also illustrated.

## 1. Introduction

**Figure 1:**
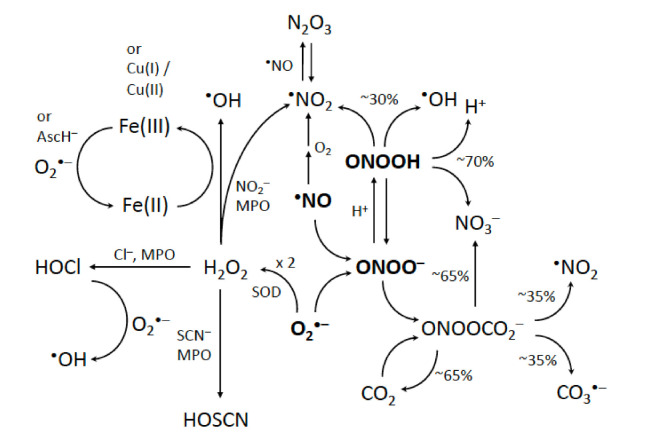
Pathways by which superoxide and nitric oxide can be converted to other oxidants in biology. The formation of peroxynitrite is emphasized. MPO, myeloperoxidase; AscH^−^, ascorbate.

Ionizing radiation produces free radicals, but damage to proteins by radicals is of much wider interest than the effects of radiation on cells and tissue. Appreciation of the involvement of free radicals in biology not arising from radiation grew dramatically following the identification of superoxide dismutase [1], a protein that catalysed disproportionation of superoxide radicals (O_2_^•−^) to form hydrogen peroxide (H_2_O_2_). One area of early interest in free-radical biology [2,3] was the possible generation of hydroxyl radicals (^•^OH) by Fenton chemistry (Fe^(II)^/H_2_O_2_) [4,5]. A dramatic expansion of the scope of free radicals in biology followed the discovery of a role in biology for peroxynitrite (peroxynitrite refers to the sum of peroxynitrite anion (ONOO^−^) and its conjugated acid, peroxynitrous acid (ONOOH, p*Ka* = 6.8)) [6,7,8,9,10] formed by the reaction of O_2_^•−^ with nitric oxide (^•^NO, nitrogen monoxide) (Figure 1), the latter initially identified to be biologically important as ‘endothelium-derived relaxing factor’ [11]. In the absence of targets, peroxynitrite rapidly decomposes to form ^•^OH and nitrogen dioxide (^•^NO_2_) radicals (reviewed in [7]). This discovery was soon followed by the realization that in the presence of bicarbonate/CO_2_, peroxynitrite could yield carbonate radicals (CO_3_^•−^) [12] and rather powerful oxidants, further extending the range of biological free-radical oxidants [12,13,14,15]. Intriguingly, CO_3_^•−^ has recently been implicated in Fenton-type chemistry [16,17].

The radiolysis of water generates three main free-radical species, as well as H_2_O_2_ and H_2_: the hydrated electron (*e*_aq_^−^), hydrogen atoms (H^•^) and ^•^OH (H_2_O^•+^ is a very short-lived precursor). All are highly reactive towards proteins. However, in this article, reflecting the wider interests noted above, we focus on outlining and illustrating the use of radiolysis methods to generate specific, individual free radicals of interest in the context of peroxynitrite biochemistry and follow their reactions with amino acids, proteins, and other biologically relevant targets. The application and versatility of pulse radiolysis to studying biochemical redox processes has been reviewed by several authors [18,19,20,21]. Herein, we aim to provide both some background discussion and specific illustrations, seeking to complement these other reviews with a focus on how radiolysis studies were adapted and utilized for the biochemical characterization of peroxynitrite-derived radicals.

## 2. Using Radiolysis to Probe Nitro-Oxidative Modifications to Amino Acids and Proteins

### 2.1. ‘Converting’ e_aq_^−^ and ^•^OH to Specific Oxidants

We focus here on oxidative damage to proteins by free radicals. While oxidation formally corresponds to electron loss, ^•^OH are not known to oxidize aromatic systems by direct electron transfer. Thus, oxidation of phenolic compounds by ^•^OH involves a two-step process: addition to the ring and then elimination of water, a reaction catalysed by acid/base and phosphate [22,23]. However, there are many other potential sites of attack of ^•^OH on proteins where hydrogen abstraction can occur. Oxidation by other biologically-important radicals, (e.g., CO_3_^•−^ and ^•^NO_2_), is more likely to involve direct electron transfer and the reactions of these oxidants or other free radicals which oxidize selectively by electron transfer have been of special interest. Saturation of aqueous solutions with nitrous oxide (~25 mM) removes *e*_aq_^−^ and generates ^•^OH radicals in <20 ns:

*e*_aq_^−^ + N_2_O (+ H^+^) → N_2_ + ^•^OH (1)

Early studies on oxidation of tryptophan (TrpH) and tyrosine (TyrOH), either free or in peptides, exploited the selectivity of secondary inorganic oxidants ((SCN)_2_^•−^, Br_2_^•−^, N_3_^•^, CO_3_^•−^, SeO_3_^•−^ and ^•^NO_2_) to oxidize these amino acids without secondary reactions such as H-abstraction from side-chains [24,25,26]. These selective oxidants are easily generated by reaction of ^•^OH with the appropriate ion (e.g., N_3_^−^ (Equation (2)), NO_2_^−^ (Equation (3)), CO_3_^2−^ (Equation (4)) or HCO_3_^−^ (Equation (5))),

^•^OH + N_3_^−^ (+ H^+^) → H_2_O + N_3_^•^ (2)

^•^OH + NO_2_^−^ (+ H^+^) → H_2_O + ^•^NO_2_ (3)

^•^OH + CO_3_^2−^ (+ H^+^) → H_2_O + CO_3_^•−^ (4)

^•^OH + HCO_3_^−^ → H_2_O + CO_3_^•−^ (5)

By using a 100-fold excess concentration ratio of N_3_^–^ to amino acid, the reaction of ^•^OH with TrpH or TyrOH is easily avoided. A notable discovery using this approach was electron transfer between these two targets in proteins [27], which has been the subject of a number of investigations (e.g., [28]).

In many cases, oxidation may be accompanied by deprotonation. Thus, the radical-cations of phenols have p*K*_a_ < 0 [29] and immediately deprotonate under physiological conditions, as does the bicarbonate radical [30]. Tryptophan radical-cations deprotonate with p*K*_a_ = 4.3 [31]. Deprotonation, either of radicals or ground state, can have important consequences on both in the pH-dependence of reduction potentials and the kinetics of electron-transfer reactions.

While, in principle, the design of such radiolysis experiments appears straightforward, there are limitations which are seldom discussed which do constrain the ‘window’ of kinetic information that can be obtained, and these are outlined below. The outstanding advantage of radiolysis as a technique to study radical reactions is that the concentrations of radicals are well-defined, and controllable, but even the radical yields need careful attention in deriving quantitative information such as extinction coefficients of species and the rate constants for radical-radical reactions.

### 2.2. Yields of Radicals Produced by the Radiolysis of Water

Confusingly, radiation chemists express yields (the amount or concentration of species produced per unit radiation dose) in different ways. The SI unit of absorbed dose is the gray (1 Gy = 1 J/kg) and yields in mol J^−1^ equate to concentrations of M Gy^−1^ (mol L^−1^ Gy^−1^) for solutions of unit density. However, before the adoption of the gray in 1975, doses were expressed either in rads (1 rad = 100 erg g^−1^ = 0.01 Gy) or eV g^−1^ (1 eV ~ 1.602 × 10^−19^ J; 1 Gy ~ 6.242 × 10^15^ eV g^−1^). Due to the cumbersome numbers, radiation chemists defined yields in terms of a *G* value, in units of molecules (100 eV)^−1^. This use is still quite common, but to add to confusion, the symbol *G* is often used for yields expressed in either way: 1 molec (100 eV)^−1^ ~ 0.1036 µM Gy^−1^ for water. We use the SI formalism here.

Radiolysis of liquid water at ambient temperatures results in ~0.5 µM ionizations per gray absorbed dose: picoseconds after the ionization event, the yield of ejected, hydrated electrons (*e*_aq_^−^) is ~ 0.48 µM Gy^−1^ [32]. However, the ionization events occur in tracks or clusters, and some species close together recombine. By the time diffusion has resulted in a homogeneous distribution of species (~0.1–0.2 µs), the yield of *e*_aq_^−^ is almost halved. It is common to summarize the species produced from water radiolysis by equations of the form:

H_2_O → *e*_aq_^−^ (0.27) + ^•^OH (0.28) + H^•^ (0.06) + H_2_O_2_ (0.070) + HO_2_^•^ (0.0008) (6)

where the yields in parentheses here are in µM Gy^−1^; the values in molec (100 eV)^−1^ are close to 10 × higher [33]. The numbers vary with different types of radiation: those above refer to irradiation of water by gamma rays or megavoltage electrons. It is important to note that the yields in Equation (6) refer only to very dilute solutions. Adding reactive chemicals or ‘scavengers’ increases the amount of water radicals scavenged since the track recombination events are intercepted. With the high concentration of scavenger in the case of N_2_O-saturated water, the yield of *e*_aq_^−^ ‘converted’ to ^•^OH by Equation (1) is ~0.33 µM Gy^−1^ rather than the value of ~0.27 µM Gy^−1^ shown in Equation (6). Further, as noted above, another common practice is again to ‘convert’ ^•^OH radicals to specific oxidizing radicals of interest, by adding high concentration of appropriate scavengers (Equations (2)–(5)), and the combination of high concentrations of scavengers of both *e*_aq_^−^ (N_2_O) and ^•^OH results in yields of secondary oxidants (e.g., by Equation (2)) which can be as high as ~0.7 µM Gy^−1^, rather than the value of ~0.55 µM Gy^−1^, the sum of the yields of *e*_aq_^−^ and ^•^OH in Equation (6). There is a useful, semi-empirical equation to predict the yields in such circumstances for different ^•^OH scavengers in N_2_O-saturated water containing an ^•^OH scavenger S, the ‘Warman-Asmus-Schuler Equation’ [33]:

[^•^OH] scavenged /µM Gy^−1^ ~ 0.54 + 0.32 (*k*[S]/*λ*^½^/(1 + (*k*[S]/λ)^½^) (7)

where *k* is the rate constant for reaction of ^•^OH with S and λ = 4.7 × 10^8^ s^−1^. Using Equation (7), if *k*[S] = 5 × 10^8^ s^−1^, then the yield of ^•^OH scavenged is ~0.70 µM Gy^−1^.

Thus, the yields of free radicals produced by irradiation of different aqueous solutions are known, reliably, to within a few percent. This is a major asset in applying radiation chemistry to study free radicals. Not only are the concentrations of radicals known, but the radicals can be produced either at a known, constant rate which can be varied by orders of magnitude simply by varying the radiation intensity, or a known concentration of radicals can be produced in a solution ‘instantaneously’ (for our purposes) using sub-microsecond pulses of radiation.

### 2.3. Measuring Rate Constants Using Radiation-Chemical Generation of Radicals: Some Limitations

Observing reactions in real time following pulse radiolysis seems at first sight to be a major advantage over inferences from final product analysis, but the method does have its limitations. Typically, doses of up to a few grays are delivered in a fraction of a microsecond. Noting the yields in Equation (6), this corresponds to producing radical concentrations in the micromolar range. Most studies use spectrophotometric detection with (typically) a time window for real-time observation of radicals of sub-microseconds to seconds. While for some specific purposes, e.g., studying spontaneous disproportionation of HO_2_^•^/O_2_^•−^ radicals, considerably higher doses/radical concentrations are used, it is generally advantageous to use the lowest dose possible. This is due to the fact that in the majority of experiments it is desired to study a reaction of a radical R^•^ with a specific solute S (Equation (8)), while minimizing unwanted contributions of radical/radical reactions (Equation (9)):

R^•^ + S → product(s) (8)

R^•^ + R^•^ → combination or disproportionation products. (9)

**Figure 2:**
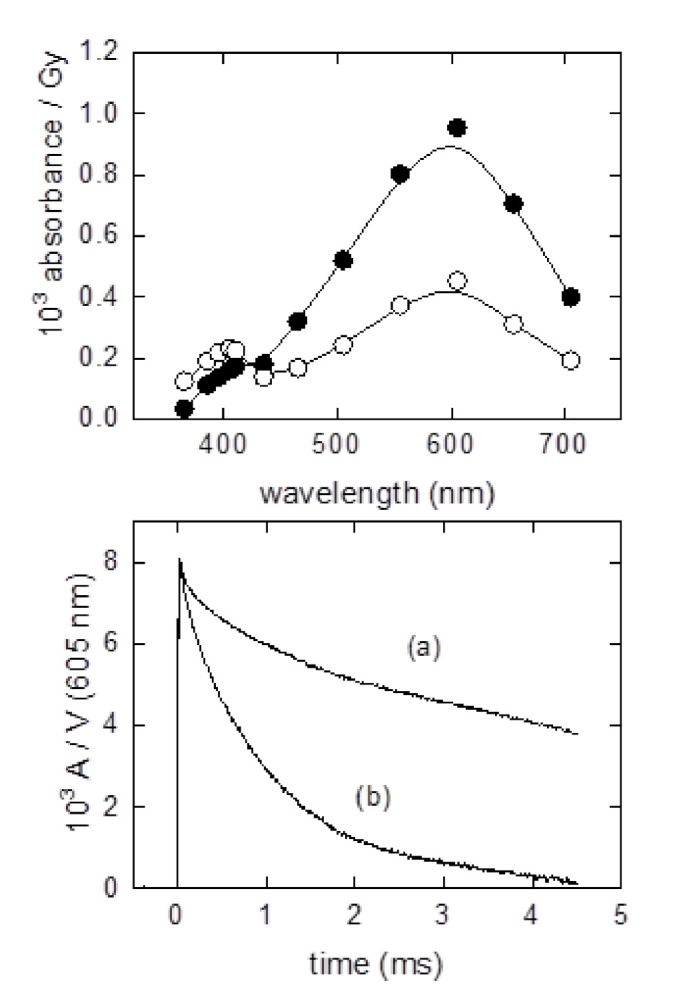
Upper: examples of transient absorption spectra of CO_3_^•–^ generated following pulse radiolysis of a solution of 40 µM *N-t-*BOC-L-tyrosine tert-butyl ester (BTBE) incorporated with liposomes prepared with egg yolk phosphatidyl choline at pH 8, 100 µs (filled circles) and 500 µs (empty circles) after a 15 Gy, 500 ns pulse (note the formation of absorption at 405 nm indicative of the formation of TyrO^•^). Lower: example transients showing decay of the absorption (A) of CO_3_^•–^; (**a**) with no BTBE and (**b**) with 40 µM BTBE present.

Since many radical/radical reactions have rate constants approaching the diffusion-controlled limit (>10^9^ M^−1^ s^−1^) the first half-life of reaction (9) might well be <500 µs even if the initial radical concentration is only (say) 1 µM. A common scenario is to use the primary water radicals (*e*_aq_^−^/^•^OH/H^•^) to generate a secondary radical (e.g., ^•^NO_2_, CO_3_^•−^or GS^•^) and monitor the reactions of the latter with the target S, reaction (8). This places limits on the concentrations of solute S, since it must be much lower than the concentration of the main radical scavenger (e.g., Br^−^, N_3_^−^, NO_2_^−^, HCO_3_^−^/CO_3_^2−^, GSH), to avoid direct reactions of the water radicals with S. In turn, limiting the concentration of S to (say) 0.5 mM to achieve this aim might mean the half-life of reaction (8) might be >140 µs (i.e., quite possibly within the same time range as reaction (9), if the rate constant *k*_5_ was as low as 10^7^ M^−1^ s^−1^; there are many reactions of interest with much lower rate constants). Thus, the design of many experiments involves careful consideration of competing reactions if reaction (8) is to dominate over reaction (9), and there are many reactions of interest which are simply too slow to be accessible due to the constraints outlined above. For the peroxynitrite-derived oxidant CO_3_^•–^ (Equations (4) and (5)) which is observed by kinetic spectrophotometry at 605 nm (Figure 2), the rate of loss of absorbance with increasing concentrations of S is used to estimate rate constants for reactions with a target S. Studying reactions of CO_3_^•−^ at physiological pH is complicated with the potential requirement of CO_2_.

Steady-state (continuous) radiolysis to generate radicals is useful in many instances. This is because at low dose rates (of the order of perhaps 1 Gy s^–1^ or lower), the steady-state concentrations of radicals R^•^ are so low that reactions of the type of (9) then contribute negligibly, and so even slow reactions (8) of specific radicals with targets can proceed with high specificity. In addition, the ability to generate radicals with a widely-varying, more biomimetic range of production rates is often of value. Even if low dose rate radiolysis can overcome problems such as reaction (9) no longer competing with (8), there may still be constraints: for example, using gamma radiolysis to oxidize TyrOH using Br_2_^•–^, while radical/radical self-reaction of the oxidant could be neglected, even 1.8 µM nitric oxide was sufficient to intercept a significant fraction of Br_2_^•–^ in competition with 500 µM TyrOH. This is so because the reactivity of NO^•^ towards Br_2_^•–^ is very much higher than reaction of the latter radical with TyrOH (see Section 4). Solubilising agents such as DMSO can cause unexpected problems. DMSO is an efficient scavenger of ^•^OH and can reduce the radical yield of ^•^OH as well as produce CH_3_^•^ radicals.

Fortunately, several thousand rate constants are readily accessible in the literature to assist with experimental design. Thus carefully-evaluated recommendations for rate constants for reactions of *e*_aq_^−^/^•^OH/H^•^ have been assembled [34,35]. Other compilations include reactions of HO_2_^•^/O_2_^•–^ [36]; peroxyl radicals [37]; phenoxyl radicals [38]; inorganic radicals [39]; aliphatic carbon-centered radicals [40]; and metal ions and metal complexes [41], all in aqueous solutions.

### 2.4. It Is Important Not to Overlook Minor Reactions

Hydrogen atoms comprise only around 10% of the water radicals in Equation (6) and their production is therefore often neglected. However, only in studying superoxide radical reactivity with oxygenated solutions containing formate to scavenge ^•^OH and generate the reductant CO_2_^•–^, where HO_2_^•^/O_2_^•–^ are the only radicals present within 1–2 µs after the end of a radiation pulse, do we encounter really ‘clean’ radical production. The ‘minor’ product H^•^ could be a significant interference, especially in oxygenated solutions due to the diffusion-controlled reaction of H^•^ with O_2_ to form superoxide.

### 2.5. Reduction (Electrode) Potentials of Radicals as a Guide to Reactivity

The rate constants of many redox reactions reflect the ‘energy gap’ quantified by the reduction potentials of the couples involved [42]. Thus, the rates of oxidation of phenols catalyzed by peroxidase enzymes have been interpreted in terms of the Marcus relationship for electron-transfer reactions [43], but there are numerous other examples where the ‘energy gap’ is reflected in the reaction kinetics [44]. Reduction potentials characterize the reaction:

*oxidant + e^−^ ⇌ reductant* (10)

and is represented by *E*(*oxidant*/*reductant*), where variants such as *E*° may define a standard potential (reactants/products with unit activity), *E*°′ to reflect quasi-standard conditions such as pH 7 when protons are involved in the couple, or subscripted with *m* and/or *i* to indicate mid-point potentials at pH*_i_* [45]. Radiation chemistry has contributed enormously to characterizing reduction potentials of couples involving radicals where either oxidant or reductant is short-lived in aqueous solution, by measuring the equilibrium constants of reversible equilibria involving the radicals and redox indicators of known electrode potential [46]. Table 1 lists some reduction potentials of couples of relevance to radiation-chemical studies of free-radical oxidative damage to amino acids at pH 7 [45]. The higher the value, the more powerful the oxidant. The bromide, azide, and thiocyanate couples are included as they have been used extensively as one-electron oxidants in model studies.

It should be noted that the reduction potentials of the amino acids will vary when incorporated in a peptide or protein, not least due to the fact that the prototropic properties may be changed from those in free solution, as well as the degree of hydration. Further, while nominally ^•^OH is the most powerful oxidant, it may react by addition/elimination reactions as noted above, or hydrogen abstraction rather than electron transfer. Of the biological peroxynitrite-derived oxidants, CO_3_^•−^ is significantly more powerful than ^•^NO_2_, and only participates in hydrogen abstraction reactions [48].

## 3. Oxidation of Tyrosine and the ‘Repair’ of the Tyrosyl Radical

TyrOH is a critical component of many proteins and is also an essential precursor to the formation of catecholamines, melanin and thyroxine, and levels of TyrOH in the body are closely regulated. It is one of the most easily oxidizable amino acids by non-enzymatic reactions. The oxidation of tyrosine residues to phenoxyl radicals (TyrO^•^), sometimes predominating as a stable amino acid radical, is essential for some enzymatic functions [49,50]. 3,3′-Dityrosine (diTyr) is also an enzymatically-controlled cross-link of TyrOH maintaining functionality of some proteins [51]. The ability of biological oxidants to generate TyrO^•^ is of much interest [14]. Importantly, TyrOH does not react directly with peroxynitrite at appreciable rates [52], but rather with peroxynitrite-derived radicals that promote its one-electron oxidation to TyrO^•^ and its subsequent evolution to 3-nitrotyrosine, diTyr and 3-hydroxytyrosine [7]. Earlier work involved studies of oxidation of TyrOH by ^•^OH and secondary oxidants such as N_3_^•^ (e.g., [27,28]). The stable products following one-electron oxidation of TyrOH depend upon the availability of antioxidants [53,54]; in physiological conditions reactions of TyrO^•^ with ascorbate (Equation (11)) can be favored over those with glutathione (GSH) (Equation (12)) [55].

TyrO^•^ + AscH^−^ → TyrOH + AscH^•^ (11)

TyrO^•^ + GSH ⇌ TyrOH + GS^•^ (12)

### 3.1. Kinetics of Reduction of Tyrosine Phenoxyl Radicals By Glutathione

**Figure 3:**
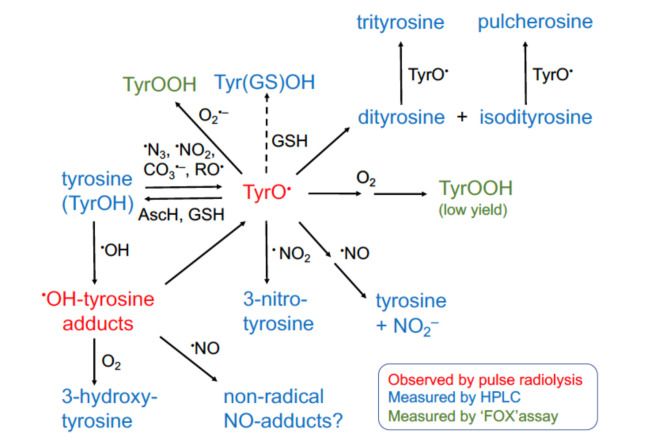
Pathways of free-radical reactions with tyrosine which can be monitored by pulse radiolysis or HPLC (with absorbance, fluorescence and mass spectrometric detection) following steady-state radiolysis. Chemical structures of products showed in the figure can be found in the following references [7,56,57,58,59,60,61]. A putative structure of a tyrosine-glutathione adducts formed from the reaction of the TyrO^•^-GS^•^ radical intermediates (dashed arrow) has been inferred from [53].

Thiols can compete with targets such as TyrOH for reaction with oxidants, but they can also ‘repair’ oxidative damage to proteins. The lifetime and fate of TyrO^•^ in biological systems is largely driven by the availability and proximity of oxidants and reductants. We investigated the reaction between TyrO^•^ radicals and GSH [53]. The formation of diTyr, from the combination of two TyrO^•^ (Equation (13)) can reflect oxidative damage; diTyr is fluorescent and thus easy to measure. Other secondary oxidation products, involving tyrosyl radicals, include isodityrosine, and the tyrosine trimers such as trityrosine and pulcherosine [56,57] (Figure 3).

3,3′-Dityrosine being a product of radical dimerization, reductive processes which ‘repair’ tyrosyl radicals can compete to decrease or eliminate the yields of diTyr. GSH reacts with TyrO^•^ with an estimated rate constant of 2 × 10^6^ M^−1^ s^−1^ pH 7.15 [53]. Figure 3 illustrates which radiolysis methods can be used to study these reactions. To determine the rate constant, TyrO^•^ were generated by pulse radiolysis and monitored by kinetic spectrophotometry at 405 nm. Proposals of a possible equilibrium existing in the ‘repair’ reaction were also investigated. By observing the fast redox equilibrium with the indicator 2,2′-azinobis(3-ethylbenzothiazoline-6-sulphonate), the reduction (electrode) potential of the (TyrO^•^, H^+^/TyrOH) couple was estimated. The mid-point reduction potential of TyrO^•^ at pH 7, *E*_m7_ (TyrO^•^, H^+^/TyrOH) = 0.93 ± 0.02 V was found to be close to that of the glutathione thiyl radical (GS^•^), *E*_m7_ = 0.94 ± 0.03 V [47]. The ‘repair’ equilibrium for the reaction of TyrO^•^ with GSH (Equation (12)) suggested that the reaction might require removal of GS^•^ (expected to be the preliminary radical product of GSH). This could occur via dimerization (Equation (14)), conjugation with thiol/thiolate (Equation (15)) or O_2_ (Equation (16)), to move the equilibrium in the direction of repair.

2TyrO^•^ → 3,3′-dityrosine (13)

GS^•^ + GS^•^ → GSSG (14)

GS^•^ + GS^−^ ⇌ (GSSG)^•−^ (15)

GS^•^ + O_2_ ⇌ (GSOO)^•^ (16)

Products from the reaction of TyrO^•^ with GSH were monitored using HPLC with absorbance, fluorescence and mass spectrometry used for detection. Oxidised GSH (GSSG) was detected following steady-state radiolysis, with the concomitant loss in the formation of diTyr with an increase in remaining GSH concentration.

However, yields of GSSG were not equal to 2 × the loss of GSH, suggesting that parallel reactions may be occurring. Indeed, evidence was seen for the formation of a conjugate of TyrOH with GSH (potentially a tyrosine thioether with GSH substituted at the 3′ position). Whether this product would be observed or be formed in a cellular system is unknown. The reaction of tyrosyl radical and superoxide radical has been reported [62] and it mainly yields tyrosyl hydroperoxide. This oxidized tyrosine derivative can be formed and be reactive in peptides and proteins in vitro [63,64,65], and possibly at sites of inflammation in vivo.

### 3.2. Kinetics of Oxidation of Tyrosine by a Model Alkoxyl Radical and Free-Radical Reactions in Hydrophobic Membranes

The formation and fate of the radical intermediates in different environments and proteins is not fully understood. The microenvironment may have a large effect on the reactivity of TyrOH and its radicals [66]. In membrane proteins, diffusion of oxidants into lipid bilayers may be hindered [67] and lateral movement of TyrO^•^ units within the membrane to allow dimerization or other conjugations to occur may be severely restricted. These physicochemical factors influencing tyrosine nitration in membranes may differ from those in aqueous solution and therefore, understanding the biochemical changes to TyrOH within a hydrophobic environment following oxidative and nitrosative stress is important [58]. Many determinants have to be taken into account, such as facilitated diffusion of ^•^NO_2_ towards membranes, exclusion of antioxidants normally present in aqueous phases such as ascorbate and GSH, solvent accessibility to tyrosine residues and presence of fatty acids which undergo lipid peroxidation reactions [68]. Tyrosine nitration and lipid peroxidation may be linked, since lipid radicals may oxidize TyrOH, fueling nitration pathways in membranes [69,70].

Incorporating TyrOH within hydrophobic compounds such as *N*-*t*-BOC L-tyrosine *tert*-butyl ester (BTBE) offers the possibility of studying aqueous micellar suspensions, liposomes, and biomembranes [66,67,69,70,71,72]. Although this presents additional constraints from scattered light in pulse radiolysis studies, extraction of products from micellar suspensions following gamma-radiolysis permits chromatographic analysis. A rate constant for reaction of lipid peroxyl radicals with BTBE has been estimated as 4.8 × 10^3^ M^−1^ s^−1^ [70]. Similar reactions with alkoxyl radicals (also intermediates in lipid peroxidation) were investigated using steady-state and pulse radiolysis [73].

The *tert*-butoxyl radical (*t*-BuO^•^) generated from radiolysis of aqueous solutions of *tert*-butyl hydroperoxide was used as a model alkoxyl radical of similar reactivity to those generated from lipid peroxides [73,74]. The reaction of *t*-BuO^•^ with TyrOH was followed by steady-state radiolysis with competition kinetics, measuring acetone by LCMS, which is produced from the β-fragmentation of *t*-BuO^•^ [73]. The concentration of TyrOH and pH of the solutions reduced the yield of acetone and the rate constant for the reaction between *t*-BuO^•^ and TyrOH was estimated as 6 × 10^7^ M s^−1^ at pH 10. The reaction was also followed by pulse radiolysis by measuring the formation of TyrO^•^ by kinetic spectrophotometry at 405 nm. The initial yield of TyrO^•^ as a function of TyrOH concentration allowed the rate constant for the reaction between *t*-BuO^•^ and TyrOH to be estimated as 7 ± 3 × 10^7^ M^−1^ s^−1^ at pH 10. As the reaction was not observable at pH 7 it was concluded that *t*-BuO^•^ preferentially oxidizes tyrosine phenolate rather than the phenol. The p*K*_a_ of phenolic hydroxyl dissociation in TyrOH is ∼10.3, which infers that at pH 7.4 the rate constant is much lower, about 3 × 10^5^ M^−1^ s^−1^. This is still significantly higher than that estimated for similar reactions by lipid peroxyl radicals [70]. However, further work is required to fully understand how lipid radicals themselves may oxidize membrane proteins in cells. Polyunsaturated fatty acids in a membrane can undergo intramolecular cyclization to epoxy-allylic radicals upon oxidation, which can combine with O_2_ to create secondary peroxyl radicals [75]. It may be that reactions with alkoxyl radicals are restricted to TyrOH residues close to the lipid-water interface; protein-derived alkoxyl radicals may have different reactivity to lipid alkoxyl radicals.

The decomposition of peroxynitrite generates a mixture of radicals at neutral pH (Figure 1). Using steady state radiolysis, radiolysis intermediates and products of TyrOH within BTBE were found to be similar to those of free TyrOH in aqueous solution (L.K. Folkes, personal communication; see Figure 2, lower panel, as well) and to those observed following reaction with peroxynitrite. However, the efficiency of nitration of TyrOH by peroxynitrite was more effective in hydrophobic compared to in aqueous conditions [66]. The yields of products (especially dimerized BTBE) were dependent upon the saturation of the lipids used to prepare the micelles, with dimerization being higher where unsaturated fatty acids were incorporated. This enhanced dimerization could be related to changes in the fluidity of the membranes (as unsaturated fatty acids create more fluidity) or through secondary reactions with lipids or lipid radicals, including competition reactions of the initiating radicals themselves with lipids. Further reactions of BTBE are discussed in Section 5.

## 4. Involvement of Nitric Oxide and Oxygen in Reactions of Tyrosyl Radicals

### The Effects of Nitric Oxide or Oxygen on the Stable Products Formed from the Tyrosine Phenoxyl Radical and Tyrosine Hydroxyl Radical Adducts

Reactions of tyrosyl radicals with nitric oxide and oxygen are illustrated in Figure 3 and [76]. The variety of radicals from the decomposition of peroxynitrite (Figure 1) complicates the determination and identification of individual radical reactions. Pulse radiolysis allows the rates of reactions of TyrOH with peroxynitrite-derived radicals to be measured, and the lifetimes of radical intermediates to be compared in the absence and presence of other reactants (such as ^•^NO or O_2_). The controlled production of tyrosyl radicals with steady state radiolysis allows yields of subsequent products, including diTyr, 3-nitrotyrosine, and 3-hydroxytyrosine (L-dopa) to be measured for individual oxidizing radicals.

Reactions of TyrOH with ^•^NO_2_ and CO_3_^•−^ proceed through the intermediate formation of TyrO^•^ which react very slowly with O_2_ [54,76] and very rapidly with ^•^NO [77]. Hydroxyl radical reacts with TyrOH through addition/elimination reactions forming ^•^OH-adduct intermediates at the *ortho* or *meta* position [78,79]. In comparison to reactions with TyrO^•^, O_2_ reacts rapidly with the C-3(^•^OH)-TyrOH adduct (*ortho*), forming L-dopa [80], whilst releasing superoxide. In the absence of, or with only low concentrations of O_2_, the *ortho* C-3(^•^OH)-TyrOH adduct can dehydrate forming TyrO^•^ [79], the same radical produced by reaction of TyrOH with NO_2_^•^ and CO_3_^•−^, in a reaction which is acid/base catalysed (including by phosphate (HPO_4_^2−^) [23,81]. In comparison, the *meta* position C-4(^•^OH)-TyrOH adduct is thought to decay through second-order reactions and does not dehydrate to form TyrO^•^ [79].

Using pulse and gamma radiolysis, it has been shown that ^•^NO_2_ reacts with TyrOH to form TyrO^•^ [26], and 3-nitrotyrosine (unique to radical reactions with ^•^NO_2_) through secondary radical-radical reactions with ^•^NO_2_. In the presence of hundreds of micromolar of O_2,_ a small decrease in the formation of these products is observed, with some evidence of low yields of hydroperoxide being formed, but no 3-hydroxytyrosine has been identified [76]. In contrast, in the presence of micromolar concentrations of ^•^NO, the loss of TyrOH is very low. Evidence exists for the probable formation of an unstable intermediate regenerating TyrOH and forming nitrite. It is important to point out that the reaction of tyrosyl radical with oxygen is rather slow, in contrast to what is observed with other amino acid derived radicals [76,82].

Hydroxyl radicals react with TyrOH through intermediate adducts, forming diTyr. In the presence of micromolar concentrations of O_2_ the yields of diTyr are reduced and 3-hydroxytyrosine is formed. In contrast, in the presence of micromolar concentrations of ^•^NO the loss of TyrOH is similar to that in the absence of ^•^NO but the yields of diTyr are extremely low [83]. There are probably intermediate non-radical ^•^NO-adducts with the ^•^OH-adducts of TyrOH. These radical-radical reactions could ‘fix’ the damage to TyrOH from reaction with ^•^OH but in doing so could alter metabolic functions of proteins or yields of metabolic compounds by altering TyrOH. Changes in the levels from expected basal concentrations of stable products resulting from TyrO^•^ can be indicative of oxidative and/or nitrative stress.

Whilst reactions of TyrOH with peroxynitrite-derived radicals can be followed by chromatography techniques, diTyr can be derived from multiple peroxynitrite-derived free-radicals and therefore may not reflect the source of the initial oxidizing radical. Also, secondary reactions producing products such as 3-nitrotyrosine are likely to be different in yield from a ‘bolus’ addition of peroxynitrite compared to ionizing radiation, especially in a heterogeneous medium. Utilizing multiple techniques can improve mechanistic understanding.

## 5. Reactions of Other Biological Targets Towards Peroxynitrite-Derived Radicals

### 5.1. Carbonate and Nitrogen Dioxide Radicals Readily React with Lipoic and Dihydrolipoic Acid

Lipoic acid (LA) and its reduced form dihydrolipoic acid (DHLA) play a major role in cellular metabolism and have redox functions [84]. Peroxynitrite reacts with LA and DHLA with second-order rate constants of 1400 and 500 M^−1^ s^−1^, respectively [85], too low for these compounds to protect against peroxynitrite-mediated damage in vivo. In contrast, peroxynitrite-derived ^•^NO_2_ reacts with LA and DHLA with rate constants of *k* = (1.3 ± 0.1) × 10^6^ and (2.9 ± 0.2) × 10^7^ M^−1^ s^−1^, respectively, and with CO_3_^•−^ (*k* = (1.6 ± 0.02) × 10^9^ and (1.6 ± 0.1) × 10^8^ M^–1^ s^–1^, respectively) [86]. Lipoic acid also reacts rapidly with ^•^OH (4.7 × 10^10^ M^−1^ s^−1^ [87]). Oxidation of LA by these radicals forms the potent one-electron oxidant LA^•+^ radical cation (*E*^0′^ LA^•+^/LA = 1.1 V) [88].

Lipoic acid is both water and fat soluble [84] and increases the oxidation efficiency of peroxynitrite-derived radicals but not the nitration efficiency. The oxidizing radicals react with LA in competition with TyrOH and the resulting LA radical cation can then oxidize TyrOH or its hydrophobic homologue causing its oxidation and dimerization [67,86]. In contrast, nitration of BTBE and TyrOH by peroxynitrite is inhibited in the presence of LA and DHLA [86]. Nitration of BTBE is through the one-electron oxidation of BTBE (from ^•^NO_2_ or CO_3_^•−^) to a phenoxyl radical which reacts in secondary reactions with ^•^NO_2_ forming 3-NO_2_-BTBE. For a bolus addition of peroxynitrite in the presence of LA, all associated radicals are generated simultaneously over a short time (half-life = 1 s). ^•^NO_2_, CO_3_^•−^ and ^•^OH can all react with LA to form LA radical cations which can oxidize BTBE to form BTBE radicals. ^•^NO_2_ may then not be available to react with BTBE radicals to nitrate them, having already reacted with the excess of LA. In contrast, in steady-state radiolysis studies no change in nitration yields of BTBE were observed in the presence of LA compared to in its absence (L.K. Folkes, personal communication). The presence of LA increased the dimerization yield of BTBE slightly compared to in its absence, which suggests that a higher yield of BTBE-TyrO^•^ are formed from reactions with the LA radicals formed from direct reaction of ^•^NO_2_ with LA. Due to the steady-state formation of ^•^NO_2_ in radiolysis studies these radicals are still able to react with resulting phenoxyl radicals before they disproportionate or dimerize, simulating a more biologically-relevant condition than fluxes of peroxynitrite-derived radicals arising from the ‘bolus’ addition of peroxynitrite [7].

### 5.2. Reactivity of Hydrogen Sulfide with Peroxynitrite

Hydrogen sulfide (here the term hydrogen sulfide includes both the undissociated (H_2_S) and deprotonated (HS^-^) species (p*K*a = 6.98)) [89] is a signaling molecule that can be generated endogenously or exogenously administered [90,91,92]. It participates in several physiological functions including blood vessel relaxation and blood pressure regulation, control of insulin secretion and in the immune system, also presenting anti-apoptosis, anti-inflammation, and anti-oxidative stress effects [93]. Peroxynitrite directly reacts with hydrogen sulfide with a rate constant of (4.8 ± 1.4) × 10^3^ M^−1^ s^−1^ (pH 7.4, 37 °C). Peroxynitrite-derived oxidants also react with H_2_S, with one-electron oxidation to a sulfanyl radical (HS^•^/S^•−^) which is an oxidizing radical (*E°’* (HS^•^,H^+^/H_2_S) = 0.91–0.94 V) [94]. Peroxynitrite addition to H_2_S at low concentrations led to oxygen consumption, which is consistent with sulfanyl radical formation followed by a radical chain reaction. The rate constant of the reaction of H_2_S with ^•^NO_2_ and CO_3_^•–^ were determined by pulse radiolysis as (1.2 ± 0.1) × 10^7^ M^−1^ s^−1^ at pH 7.5 (25 °C) and (2.0 ± 0.3) × 10^8^ M^−1^ s^−1^ at pH 7 (20 ± 2 °C) [94]. Hydrogen sulphide also reacts with several two-electron oxidants such as hydrogen peroxide, hypochlorite, and taurine chloramine, with rate constants of 0.73 ± 0.03, (8 ± 3) × 10^7^, and 303 ± 27 M^−1^ s^−1^, respectively (pH 7.4, 37 °C). The protective effects of H_2_S, which are present in tissues at low micromolar or submicromolar concentrations, are probably not completely dependent on direct reactions with oxidants.

## 6. Reactivities of Peroxynitrite-Derived Radicals with a Spin Trap and an Iron Chelator

### 6.1. Reactions of CO_3_^•−^ and NO_2_ with DMPO

‘DMPO’ (5,5-dimethyl-1-pyrroline-*N*-oxide) is widely used as a ‘spin trap’ for radicals involving electron paramagnetic resonance (EPR) detection, since the radical-adducts formed can be long-lived and the signals characteristic of specific radicals. However, for such radical scavengers to be used with confidence, it is important to characterize their reactivities towards key radicals. CO_3_^•−^ reacts with DMPO leading to DMPO-OH formation. Pulse radiolysis, EPR, and steady-state competition methods were used to estimate the reactivity of CO_3_^•−^ with DMPO [95]. The rate constant of the reaction was 2.5 × 10^6^ M^−1^ s^−1^. Accordingly, DMPO inhibited tyrosine oxidation to diTyr by CO_3_^•−^ produced by a Fenton system composed of Fe^(II)^-DTPA plus H_2_O_2_ in the presence of HCO_3_^−^. However, DMPO-OH yields decreased when using this Fenton system plus HCO_3_^−^ compared with no addition. This is attributed to CO_3_^•−^ consumption through reaction with Fe^(II)^-DTPA, with a rate constant of 6.1 × 10^8^ M^−1^s^−1^. The reaction of DMPO and CO_3_^•−^ contributes to DMPO-OH formation in cellular systems, as shown using macrophages stimulated to produce peroxynitrite extracellularly in the presence of HCO_3_^−^. DMPO reacts with ^•^NO_2_ yielding an adduct which, according to mass spectrometry analysis, is consistent with the structure DMPO-ONO [96]. Importantly, early spin-trapping experiments characterizing peroxynitrite homolysis found that excess ^•^NO_2_ destroyed the DMPO-OH signal [97].

### 6.2. Reactions of CO_3_^•−^ and ^•^NO_2_ with Desferrioxamine

As noted earlier, initial interest in free radicals in biology focused on the role of Fe^(II)^ and Fenton chemistry to generate ^•^OH. A common method to assess such involvement is based on the use of the iron chelator, desferrioxamine [3], but again it is important to assess whether the chelator itself reacts with other radicals (revealed by radiolysis and other kinetic methods) [98]. Desferrioxamine B is a hexadentate trihydroxamic acid siderophore that binds tightly and in a 1:1 ratio with ferric iron (*K*_d_~10^−31^ M), which is not able to participate in Haber-Weiss mechanisms [99]. It protects biomolecules from oxidative damage through iron chelation-dependent and -independent mechanisms both in vitro and in vivo [100]. The mechanisms that are iron chelation-independent most frequently involve the reaction of desferrioxamine with oxidizing species such as hydroxyl and superoxide radicals [101,102].

Desferrioxamine inhibits peroxynitrite-mediated oxidations. However, peroxynitrite does not directly react with desferrioxamine [98]. The protective actions of desferrioxamine on peroxynitrite-mediated oxidations depend on its ability to react with peroxynitrite derived radicals, ^•^OH, CO_3_^•−^, and ^•^NO_2_, forming the relatively-stable desferrioxamine nitroxide radical. Accordingly, desferrioxamine decreases peroxynitrite-dependent tyrosine nitration and dimerization in the absence as well as in the presence of CO_2_. Furthermore, it also decreases peroxynitrite-dependent light emission in carbonate buffer pH 10 (ascribed to CO_3_^•−^ formation). Using pulse radiolysis, the rate constants of the reactions of desferrioxamine with CO_3_^•−^ and ^•^NO_2_ were determined as (1.70 ± 0.02) × 10^9^ M^−1^ s^−1^ and (7.6 ± 0.4) × 10^6^ M^−1^ s^−1^ at pH 7.5, respectively [98]. Furthermore, TyrO^•^ also oxidizes desferrioxamine, with a rate constant of (6.3 ± 0.2) × 10^6^ M^−1^ s^−1^ at pH 7.4, while the nitroxide radical of desferrioxamine is able to oxidize thiols such as GSH as well as protein thiols. Thus, the actions of desferrioxamine as an antioxidant can be complex, and kinetic information provided by pulse radiolysis allows to further rationalize its effects.

## 7. Conclusions

A common misconception is that radiation produces such complex fragmentation of molecules that it is of interest only to specialists. In fact, the radiolysis of water is extremely well understood both qualitatively and quantitatively. Many specific free radicals can be generated by exploiting this knowledge and radiolysis can thus be applied to studying diverse reactions of biological interest involving free radicals produced in both normal and pathological conditions in the absence of radiation. Some studies, such as those observing radicals directly in real time following a microsecond pulse of radiation, do require specialist equipment. However, much can be achieved using continuous radiation from sealed or machine sources that are much more accessible. A huge literature concerning rate constants for radical reactions has been built up. We have illustrated here how this experience can be applied in the wider field of free radicals in biology. In particular, following on from the early work of Prütz et al. [26,27], we have focused on how we (and others) have utilized radiation chemistry to study some of the peroxynitrite-derived radical reactions with tyrosine and other biological targets, helping to rationalize its biological effects. In this review, we have addressed here only a part of the extensive work that had focused on oxidative modifications to tyrosine (Figure 3). The reader is referred to an authoritative recent review which illustrates both the importance and complexity of this area [103]. Utilizing multiple techniques, including radiation chemistry, can only add further mechanistic understanding in the areas of redox and free radical biology and biomedicine.

## Figures and Tables

**Table 1:** Reduction (electrode) potentials of biologically relevant redox pairs at pH 7 *^a^*.

Couple (Oxidant/Reductant)	*E*°′/V
^•^OH, H^+^/ H_2_O	2.32
Br_2_^•−^/ 2 Br^−^	1.63
CO_3_^•−^/ CO_3_^2−^	1.57
N_3_^•^/ N_3_^−^	1.33
(SCN)_2_^•−^/ 2 SCN^–^	1.30
^•^NO_2_/ NO_2_^−^	1.04
Trp^•^, H^+^/ TrpH *^b^*	1.03
GS^•^, H^+^/ GSH *^c^*	0.94
TyrO^•^, H^+^/ TyrOH *^d^* LOO^•^, H^+^/ LOOH LO^•^, H^+^/ LOH HS^•^, H^+^/ H_2_S	0.91 1.01 1.76 0.93

*^a^* Ref. [45]; typical uncertainty ± 0.02 V. *^b^* TrpH, tryptophan; *^c^* GSH, glutathione, ref. [47]; *^d^* TyrOH, tyrosine.
